# High‐Throughput Ellipsometric Contrast Microscopy of Lateral 2D Heterostructures for Optoelectronics

**DOI:** 10.1002/smtd.202500437

**Published:** 2025-07-09

**Authors:** Teja Potočnik, Oliver Burton, Suman K. Chakraborty, Purbasha Ray, Ralf Mouthaan, Peter J. Christopher, Zeinab Tirandaz, Xiaofan Lin, Hannah J. Joyce, Stephan Hofmann, Prasana K. Sahoo, Jack A. Alexander‐Webber

**Affiliations:** ^1^ Department of Engineering University of Cambridge 9 JJ Thompson Avenue Cambridge CB3 0FA UK; ^2^ Materials Science Center Indian Institute of Technology Kharagpur Kharagpur West Bengal 721302 India; ^3^ Centre of Light for Life University of Adelaide North Terrace Adelaide SA 5005 Australia; ^4^ Department of Engineering University of Nottingham Nottingham NG7 2RD UK

**Keywords:** 2D materials, computer vision, ellipsometric contrast microscopy, high‐throughput characterization, lateral heterostructures, optoelectronic devices

## Abstract

Covalently‐bonded lateral 2D heterostructures offer unique (opto)electronic functionalities and can be deposited during a single growth process. However, the position of lateral junctions is typically uncontrolled due to random nucleation processes, which necessitates post‐growth identification of suitable heterojunction regions for device integration. Here, ellipsometric contrast microscopy (ECM) is demonstrated to evaluate 2D lateral monolayer MoSe_2_‐WSe_2_ and MoS_2_‐WS_2_ heterostructures, which enables rapid imaging with high material‐contrast down to sub‐nanometer thickness for high‐throughput characterisation of heterostructure domains. In addition, a computer vision algorithm provides precise identification of individual monolayer heterostructure junctions and their integration into rectifying devices and photodetectors. These results establish the advantages of ECM for reliable, fast characterization and large‐scale integration of atomically thin 2D heterostructures into advanced optoelectronic devices, with potential extension to other nanomaterials.

## Graphical abstract

Ellipsometric contrast microscopy (ECM) is demonstrated to evaluate 2D lateral monolayer MoSe_2_‐WSe_2_ and MoS_2_‐WS_2_ heterostructures, which enables rapid imaging with high material‐contrast down to sub‐nanometer thickness for high‐throughput characterisation of heterostructure domains. These results establish the advantages of ECM for reliable, fast characterization and large‐scale integration of atomically thin 2D heterostructures into advanced optoelectronic devices.

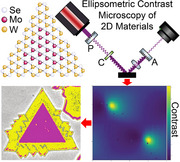

## 1 Introduction

Semiconducting 2D transition metal dichalcogenides (TMDs) have been extensively explored for electronic applications, owing to diverse properties such as structural stability, absence of dangling bonds, and high mobilities.^[^ ^1^ ^]^ In addition, the bandgap of several 2D TMDs can be tuned – by varying their layer number, doping and strain – from visible to near infra‐red regions of the electromagnetic spectrum,^[^ ^2^, ^3^, ^4^ ^]^ suitable for optoelectronic applications,^[^ ^5^, ^6^ ^]^ Prominent 2D TMD examples include MoS_2_, WS_2_, MoSe_2_ and WSe_2_, which have been widely studied for transistor,^[^ ^7^, ^8^, ^9^, ^10^ ^]^ and photodetector,^[^ ^11^, ^12^, ^13^, ^14^, ^15^ ^]^ applications. The properties of 2D TMDs are layer number‐ and doping‐dependent,^[^ ^5^, ^6^ ^]^ The integration of distinct monolayer TMDs into both vertically and laterally assembled structures introduces novel functionalities and avenues for manipulating their optical and electrical properties.^[^ ^16^ ^,^ ^17^ ^]^

Interactions at the interface between two materials can be manipulated by lattice structure matching, rotation or external fields.^[^ ^16^ ^]^ While vertical integration of mechanically exfoliated 2D materials,^[^ ^17^, ^18^, ^19^, ^20^ ^]^ offers flexibility in combining any number of TMDs and other materials, scalability and interface contamination challenges persist in the 2D transfer process.^[^ ^16^ ^]^ Furthermore, strong interlayer coupling in vertical heterostructures can significantly modify the electronic and optical properties of the individual layers, such as altering exciton binding energies, inducing interlayer charge transfer, and shifting band alignments. In contrast, TMD lateral heterostructures,^[^ ^21^, ^22^, ^23^, ^24^, ^25^, ^26^ ^]^ offer distinct advantages. They enable the creation of atomically sharp interfaces without transfer‐induced contamination or interlayer interactions, for the formation of well‐defined lateral junctions. The in‐plane geometry allows for easier integration with existing planar fabrication techniques.

Most recently, lateral heterostructures have been fabricated using sequential edge epitaxy, by changing the composition of the reactive gas environment in the presence of water vapor, allowing in situ control of individual flake growth.^[^ ^27^ ^]^ Advancements in the growth processes of heterostructure materials not only enable the exploration of complex material systems for optoelectronics but also extend to applications such as memristors and biosensors,^[^ ^16^, ^28^, ^29^, ^30^ ^]^

Although the growth of lateral heterostructures can be highly controlled, efficient integration of these materials into device applications remains a challenge. 2D TMD domains typically remain randomly distributed on a substrate, requiring identification and/or transfer of individual flakes, which is a time‐consuming and manually‐intensive process that limits research into complex 2D heterostructure‐based devices. To address this issue, there has been a notable shift toward automation in material feature recognition from microscopic images. This trend is primarily driven by advancements in computer vision, machine learning, and deep learning techniques,^[^ ^31^, ^32^, ^33^, ^34^, ^35^, ^36^, ^37^ ^]^ supported by innovations in microscopy hardware automation,^[^ ^38^, ^39^, ^40^, ^41^ ^]^

The structural similarities of TMDs enable them to be grown in the same process, but this close resemblance poses a challenge for identification and characterization using standard techniques, including computational image processing methods. Multiple techniques are often required to be able to characterize individual TMD materials within a heterostructure and accurately identify the interfaces between them. Most commonly, Raman spectroscopy,^[^ ^6^, ^27^, ^39^, ^40^ ^]^ photoluminescence spectroscopy,^[^ ^6^, ^27^, ^39^, ^40^, ^41^ ^]^ scanning electron microscopy (SEM),^[^ ^24^, ^27^, ^39^, ^42^ ^]^ and transmission electron microscopy,^[^ ^22^, ^24^, ^27^, ^39^, ^42^ ^]^ are used for heterojunction characterization. However, these approaches are often time‐consuming and may depend on the choice of substrate, which might not be universally compatible with all fabrication processes. For example, Raman spectroscopy operates as a point‐by‐point technique, which limits its speed, while SEM is a low‐throughput method that can introduce charging effects.

Ellipsometry, on the other hand, is an alternative that measures the change in polarization of light upon reflection from the sample, and serves as a valuable method for determining the physical properties of 2D materials.^[^ ^43^ ^]^ Ellipsometry measures the ratio of the reflection coefficients for the p and s polarization of light (*R_p_* and *R_s_* respectively) given by *ρ *= *R_p_/R_s_* = tan(*Ψ*)e^i^ *^Δ^*, where *Ψ* is the amplitude ratio of p‐polarized light to s‐polarized light, and *Δ* is the phase difference between the p‐ and s‐polarized components of the reflected light. To determine the full complex dielectric function and thickness of the material, wavelength‐dependent *Ψ* and *Δ* need to be fitted to an optical model.^[^ ^43^ ^]^

Spectroscopic imaging ellipsometry uses the same principles with the addition of a CCD detector, enabling wide‐field imaging rather than point measurements or those taking averages of large areas. Spectroscopic ellipsometry measures *R_p_* and *R_s_* as a function of wavelength (*λ*), angle of incidence (AOI) and polarizer (P), analyzer (A) and compensator (C) angles with spatially resolved data. This allows for the simultaneous characterization of optical properties over localized regions with a lateral resolution of ≈1 µm. It is substrate agnostic and can provide thickness information with single‐atomic layer precision,^[^ ^44^, ^45^ ^]^ making it suitable for a range of heterogeneous surfaces. Related techniques such as surface‐enhanced ellipsometric contrast microscopy have been effective for high‐speed thin‐film analysis by enhancing materials’ contrast using carefully designed interference effects in multi‐layer substrates.^[^ ^46^ ^]^ While effective, this reliance on tailored substrates can limit applicability. Therefore, high‐speed, substrate‐agnostic, snapshot imaging techniques are essential for improved efficiency and accuracy of nanostructured material characterization.

Alternatively, ellipsometer settings (AOI, P, A, C) can be optimized to obtain maximum material and thickness contrast before being fixed for the duration of imaging. This method, called ellipsometric contrast microscopy (ECM),^[^ ^47^, ^48^, ^49^, ^50^, ^51^ ^]^ measures the intensity of light rather than ellipsometric angles *Δ* and *Ψ*, making it significantly faster if some prior knowledge of the sample is assumed,^[^ ^47^, ^51^ ^]^ The versatile control of the imaging conditions allows for high‐throughput high‐contrast imaging with single atomic layer thickness sensitivity on a wide range of substrates, including metal foils, Si wafers without thermally grown oxide layers, and 2D van der Waals vertical heterostructures, where conventional brightfield reflection optical microscopy imaging achieves negligible contrast,^[^ ^47^, ^51^ ^]^

Here, we introduce ECM to identify and characterize MoSe_2_‐WSe_2_ and MoS_2_‐WS_2_ lateral heterostructures. We demonstrate that ECM provides high material contrast of 2D lateral heterostructures under optimized imaging conditions, facilitating the identification, characterization and fabrication of simple diodes on individual heterojunctions. Heterojunction diodes exhibit a photocurrent at source–drain bias *V_DS_* = 0 V which is indicative of a photovoltaic effect in the p–n junction. These findings show high throughput spatially resolved characterization of complex 2D materials and provide a scalable approach toward their integration into practical applications.

## 2 Results and Discussion

**Figure 1:**
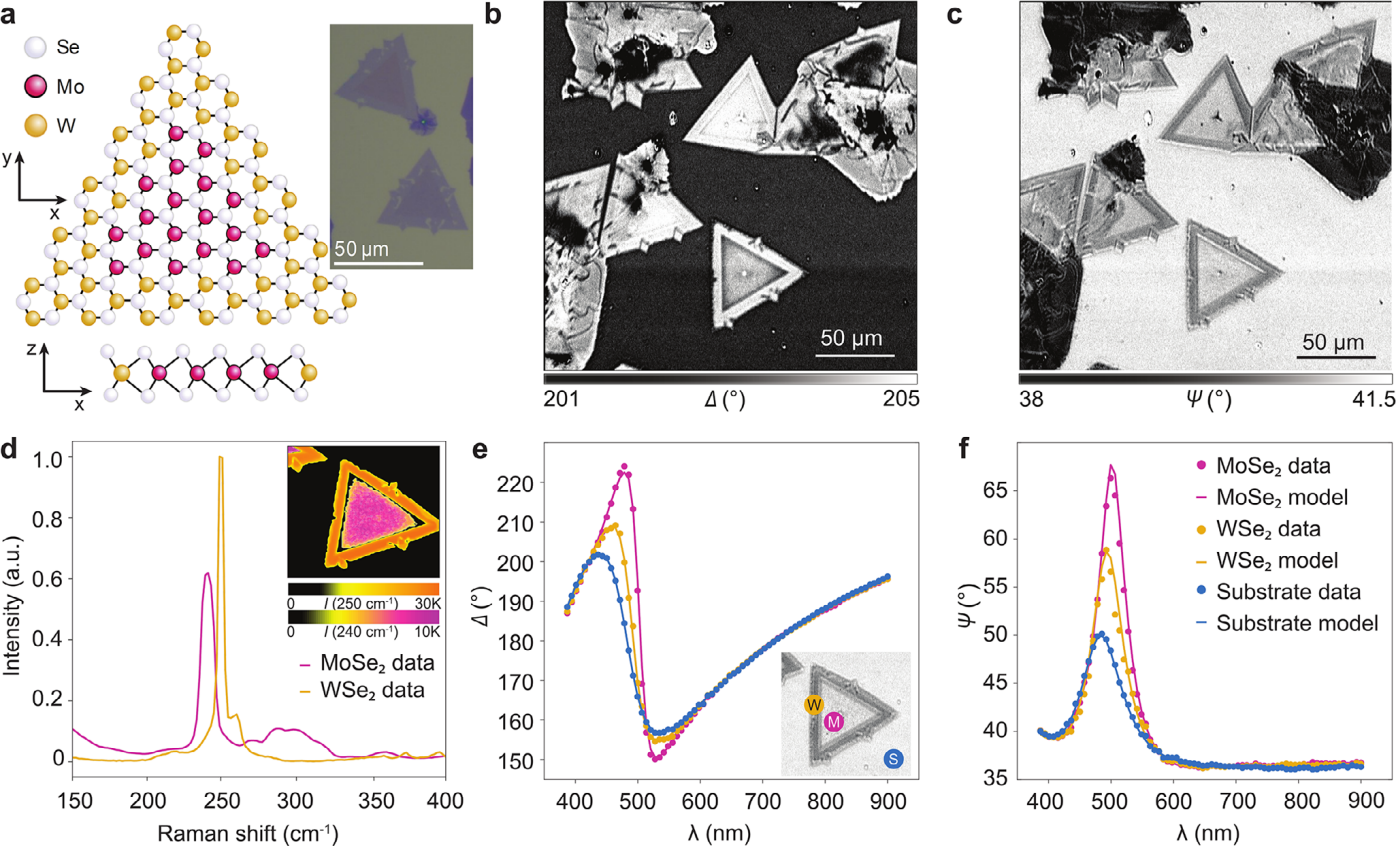
a) Schematic atomic model and optical microscopy (inset) of lateral MoSe_2_‐WSe_2_ heterostructures on SiO_2_/Si. b) Example Δ and c) Ψ maps of MoSe_2_‐WSe_2_ heterostructures at 436 nm measured in rotating compensator ellipsometry (RCE) imaging mode. d) Raman spectra of MoSe_2_ and WSe_2_ regions from the same heterostructure region. Inset: Intensity maps at the Raman peaks of 250 and 240 cm^−1^ corresponding to MoSe_2_ and WSe_2_, respectively. The color bars show counts at 250 and 240 cm^−1^. e) Δ and (f) Ψ as a function of wavelength for the bare oxidized silicon (S) substrate, WSe_2_ (W) region, and MoSe_2_ (M) regions, averaged over a continuous area, shown in the inset of (e). Solid lines represent the fitted model.

MoSe_2_‐WSe_2_ samples were grown on SiO_2_/Si substrates using a one‐pot sequential‐edge epitaxy synthesis method, with WSe_2_ growing around the edge of central MoSe_2_ islands (**Figure** **1**a).^[^ ^27^ ^]^ 2D TMDs show significant reaction anisotropy, including lateral dependence on the edge termination.^[^ ^52^ ^]^ This leads to geometrically distinct domain shapes, which present here as approximately equilateral triangles.^[^ ^52^ ^]^ Isolated triangular flakes are observed in the optical micrograph of Figure 1a, but the interface between the MoSe_2_ and WSe_2_ is barely perceptible in reflected light optical microscopy under white light illumination.

We initially perform imaging rotating compensator ellipsometry (RCE) on MoSe_2_‐WSe_2_ heterostructures. P and A were fixed at 45°, and the AOI at 41°. The wavelength was swept from 380 to 900 nm in 7 nm steps, acquiring a total of 75 spatial *Δ* and *Ψ* maps. Examples of *Δ* and *Ψ* at *λ* = 436 nm are shown in Figure 1b,c.

To validate the subsequent model fitting of the ellipsometry data, we also perform Raman spectroscopy on the same MoSe_2_‐WSe_2_ flake. Raman spectra are shown in Figure 1d corresponding to regions of interest as indicated in the inset of Figure 1e. The Raman spectra show the characteristic peak at *A_1g_* peak at ≈ 240 cm^−1^ for MoSe_2_, located in the centre of the heterostructure, and the *E^1^_2g_* peak at ≈ 250 cm^−1^ for WSe_2_, located around the edge of the heterostructure. These values are consistent with the monolayer interpretation for each region.^[^ ^53^, ^54^ ^]^

For model fitting, three regions of the ellipsometry data were chosen; S) the substrate, M) the inner region of the flake, MoSe_2_, and W) the outer region of the flake, WSe_2_ as indicated in the inset of Figure 1e. The average *Δ* and *Ψ* were determined for each region as a function of wavelength, as shown in Figure 1e,f. The SiO_2_/Si model from the Accurion EP4 software was fitted to and used to determine the thickness of SiO_2_ as t_SiO2_ = 276.7 nm. The SiO_2_ thickness was then fixed when subsequently fitting to models of MoSe_2_ and WSe_2_ materials.

The dielectric functions of MoSe_2_ and WSe_2_ were modelled using the Tauc‐Lorentz function (see Methods in Supporting Information, SI) and are shown in SI Figure S1 (Supporting Information). The thicknesses of MoSe_2_ and WSe_2_ were calculated to be t_MoSe2_ = 0.69 nm and t_WSe2_ = 0.62 nm. The theoretical monolayer thickness is 0.65 and 0.66 nm for MoSe_2_^[^ ^55^ ^]^ and WSe_2_^[^ ^56^ ^]^ respectively, and so it is concluded that these materials are both monolayer. Deviations from the theoretical thickness can be a result of variations in chemical interaction between the materials and the substrate for different growth and processing conditions.

**Figure 2:**
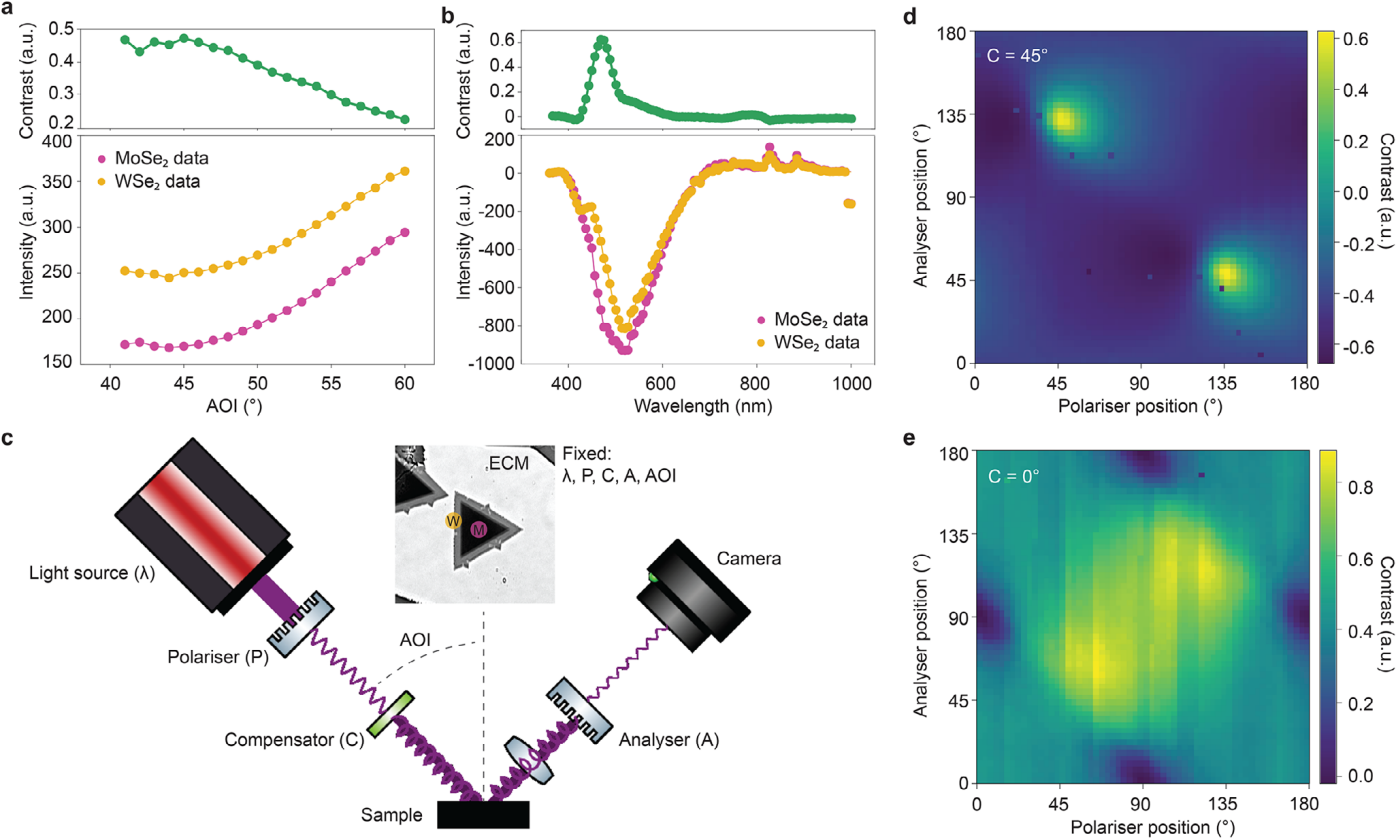
Optimization of ellipsometry parameters to maximize contrast. a) Calculated Weber contrast between MoSe_2_ and WSe_2_ regions and intensities of MoSe_2_ and WSe_2_ regions (ECM image shown inset of (c)) as a function of AOI. b) Calculated Weber contrast between MoSe_2_ and WSe_2_ regions and intensity of MoSe_2_ (M) and WSe_2_ (W) regions (shown in the inset of (c)) as a function of wavelength with the intensity of the bare substrate subtracted. c) Schematic of the ellipsometer setup. Inset shows the regions from the image with optimized settings, AOI = 41° and wavelength of 475 nm. d,e) Contrast as a function of analyzer and polarizer position for compensator fixed at (d) 45° and (e) 0°.

To maximize the contrast between MoSe_2_ and WSe_2_ using ECM we optimized the ellipsometry settings as shown in **Figure** **2**. The image contrast between the two materials was calculated using the Weber contrast relation: (*I_material1_* ‐ *I_material2_*)/(*I_material2_*), where *I_material1_* and *I_material2_* are the average pixel intensity values of the two different TMD materials. Figure 2a shows the measured intensity and contrast as a function of AOI, with the calculated contrast for the regions of interest shown in the inset of Figure 2c. The AOI was varied from 41° to 60° at P = 0°, A = 0° and C = 0°, and at a fixed wavelength, λ = 475. The maximum contrast is seen at AOI less than 50°, with the minimum AOI (41°) limited by the geometrical constraints of the ellipsometry system. The highest contrast was found at AOI 41° and 45°. We fix the AOI at 41°, as this value also corresponds to a higher intensity, and then measure the intensity as a function of wavelength from 350 to 1000 nm. Figure 2b shows the calculated contrast between the averaged inner (MoSe_2_) flake region and the outer (WSe_2_) flake region, as shown in the inset. The wavelength at which we observe the strongest contrast was found to be 475 nm. Further examples are shown in Figure S2 (Supporting Information).

The schematic of the ellipsometer setup is shown in Figure 2c. To further optimize the ellipsometric parameters for maximum contrast, we measure the contrast at AOI = 41°, and λ = 475 nm as a function of P and A at two different C angles, C = 45° and C = 0° (Figure 2d,e, respectively). For C = 0°, it can be seen that the maximum contrast is achieved at P = 60°, A = 60° (Figure 2e), and for C = 45°, contrast is maximum around P = 55°, A = 140° (Figure 2d). Based on these optimizations, the ellipsometer parameters were fixed at AOI = 41°, λ = 475 nm, C = 45°, P = 55°, and A = 140° to map the entire sample surface with high material contrast.

**Figure 3:**
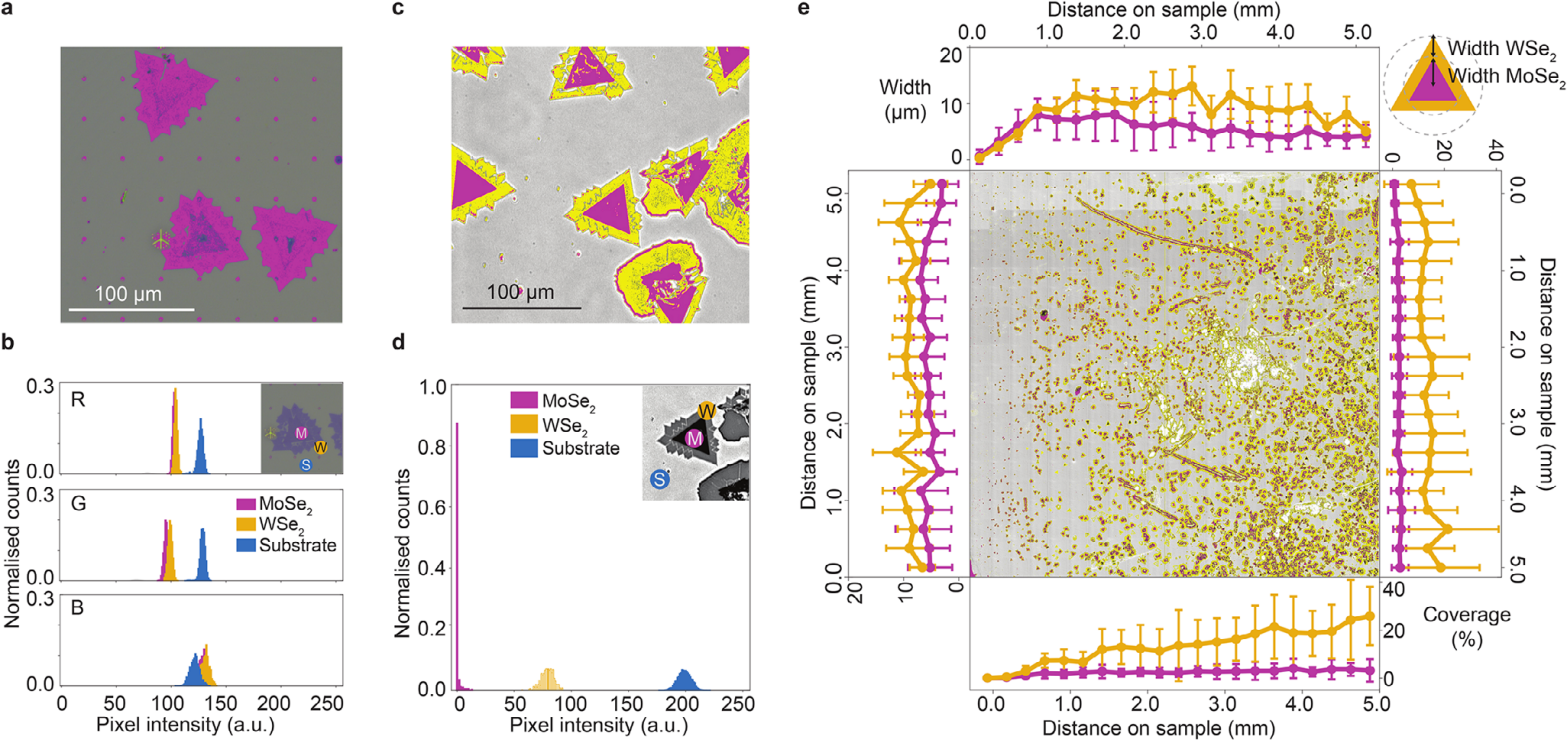
a) Image segmentation used to detect MoSe_2_ material from the optical micrograph of MoSe_2_‐WSe_2_ heterostructures. b) Histogram of an RGB image shown in (a) for R, G, and B pixels of i) MoSe_2_, ii) WSe_2_ and iii) substrate regions as shown in the inset. c) Image segmentation used to detect MoSe_2_ material from an ellipsometry image at optimized contrast settings of MoSe_2_‐WSe_2_ heterostructures. d) Histogram of a grayscale ECM image shown in the inset for pixels of MoSe_2_ (M) WSe_2_ (W) and substrate (S) regions, where >1500 pixels were sampled for each of the regions, indicated in the inset e) High‐throughput ECM map at optimized contrast and computer vision segmentation of MoSe_2_ (pink) and WSe_2_ (yellow) flakes. The plots on the sides show extracted material properties from detected heterostructure flakes, showing coverage (bottom and right) and approximate center‐to‐edge (MoSe_2_) and edge‐to‐edge (WSe_2_) width (top and left), also shown in the inset.

**Figure** **3**a shows a reflected light optical microscope image of a lateral heterostructure sample under white light illumination and an attempt at segmenting the image to detect inner (MoSe_2_) material only. The image processing algorithm, based on RGB intensity thresholding detected the outer flake area (WSe_2_) as well, which is not helpful for characterization of individual TMD materials or isolating the interfaces between them. Figure 3b shows the histograms for each of the RGB pixels, showing the frequency of each pixel intensity averaged for the regions of interest shown in the inset. We can see that regions of MoSe_2_ and WSe_2_ have similar pixel intensity for each of the three channels, as their respective histograms overlap and are therefore challenging to distinguish consistently by standard image segmentation processes.

On the other hand, contrast‐optimized ECM images indicate a clear distinction between MoSe_2_ and WSe_2_, with both materials being easy to identify using image segmentation (Figure 3c). Figure 3d shows a histogram of intensities from a grayscale ECM image, with the pink (MoSe_2_) and yellow (WSe_2_) regions isolated. This means that a simple intensity threshold can be applied to reliably segment the three regions from within the image. With this, it is now possible to quickly map large areas, and determine the material properties. Fast characterization of as‐grown materials is crucial to providing feedback for optimizing growth efforts, and important characteristics such as coverage, nucleation density, and radial growth rate can be inferred.

We apply this technique to the entire 5 mm × 5 mm sample, and obtain a high‐resolution map with segmented regions of the two TMD materials (Figure 3e) with MoSe_2_ regions colored in pink and WSe_2_ in yellow. Using a combination of ellipsometry and computer vision, we can identify each individual material. From the image shown in Figure 3e, we identified over 1300 individual heterostructure flakes. The graphs on the bottom and right‐hand side of Figure 3e show statistical information on each material's coverage as a function of position on the sample. This was calculated using the average area of each TMD material over the area of the substrate. We can see a more uniform distribution of MoSe_2_ than WSe_2_, with a significantly higher coverage at the bottom right corner of the sample. The graphs on the top and left‐hand side of the image show the approximate average center‐to‐edge (MoSe_2_) and edge‐to‐edge (WSe_2_) width of the flake of each material as a function of position on the sample, which is proportional to the average lateral growth extents in a particular region of the sample. We observe that the sizes of the MoSe_2_ and WSe_2_ regions are proportional to one another, with the MoSe_2_ occupying about one third and WSe_2_ occupying two thirds of each flake's area. This indicates that the lateral growth rates of the two materials are approximately proportional, and equally influenced by position within the growth reactor. Thus, the presented methodology provides insight for understanding the complexities of growth processes in heterostructure materials. Other techniques, such as SEM or Raman, can provide achieve identification of TMD heterostructure regions, but are lower‐throughput therefore not scalable to large sample areas. The benefits of ECM over other techniques are highlighted in Table S2 (Supporting Information). Other studies have demonstrated the characterization of h‐BN and graphene heterostructures, including layer number and defect density,^[^ ^47^ ^]^ as well as the characterization of twist angle from twisted bilayer graphene^[^ ^51^ ^]^ using ECM. Contrast‐optimized ECM mapping provides accurate information on complex materials, with reliable material‐dependent segmentation, while only taking a couple of minutes to map an entire chip of area of 25 mm^2^. While we have optimized ECM settings here to maximize contrast between materials, imaging conditions can similarly be optimized for layer‐number contrast within a single material (Figure S3, Supporting Information), demonstrating the versatility of the technique.

It has been shown that a junction between MoSe_2_ (n‐type) and WSe_2_ (p‐type) intrinsically forms a p–n junction with a strong built‐in potential,^[^ ^29^, ^40^, ^57^ ^]^ The device performance is determined by the band alignment and the height of the built‐in potential. Under photoexcitation at zero bias we could expect to observe the photovoltaic effect, which is relevant to energy‐efficient self‐powered photodetectors. Here we use our ECM methodology to identify heterostructure domains on which we fabricate MoSe_2_‐WSe_2_ p–n junction devices.

**Figure 4:**
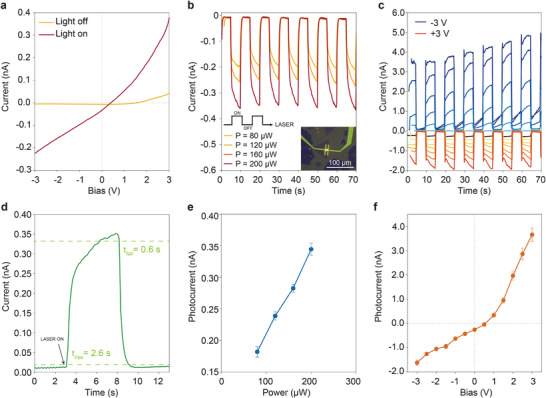
a) *I–V* curve of MoSe_2_‐WSe_2_ heterojunction device under dark and white light measurement conditions. b) Time‐response of MoSe_2_‐WSe_2_ heterojunction device under modulated laser excitation at 685 nm wavelength and V_DS_ = −1 V for different laser power settings: 80, 120, 160, and 200 µW. The laser spot is shown in the inset. (c) Time‐response of MoSe_2_‐WSe_2_ heterojunction device under pulsed incident light at 685 nm wavelength and laser power of 200 µW at different V_DS_ values ranging from −3 to 3 V in 0.5 V increments. d) Time‐response of MoSe_2_‐WSe_2_ heterojunction device showing a rise and fall time of 2.6 s and 0.6 s, respectively. e) MoSe_2_‐WSe_2_ heterojunction device's photoresponse as a function of laser power at V_DS_ = −1 V. The error bars represent the standard deviation of the photoresponse data calculated for each pulse within the 70 s timeframe. f) MoSe_2_‐WSe_2_ heterojunction device's photoresponse as a function V_DS_ bias at laser power of 200 µW. The error bars represent the standard deviation of the photoresponse data calculated for each pulse within the 70 s timeframe.

Using machine readable markers patterned lithographically on the chip,^[^ ^38^ ^]^ we register the locations of heterostructure flakes identified from ECM maps and fabricate heterojunction devices with Ti/Au contacts, each having one terminal on the MoSe_2_, and the other on the WSe_2_ (5 µm channel length and 40 µm channel width, **Figure** **4**).

Figure 4a shows the *I–V* characteristics of the devices, measured in dark conditions and under white light global illumination from a halogen lamp. We also perform time‐response measurements using a λ = 685 nm laser spot of ≈ 4 µm illumination on the heterostructure junction, positioned in the middle of the channel of the device (inset of Figure 4b). At fixed *V_DS_* = −1 V, we pulse the laser spot illumination approximately every 10 s (5 s on, 5 s off) and measure the photocurrent *I* for different laser power settings: 80, 120, 160, and 200 µW (Figure 4b). Additionally, we measure the current as a function of time over multiple laser pulses at 200 µW and different *V_DS_* values ranging from −3 V to +3 V (Figure 4c).

The time response of the device shown in Figure 4b is shown again in Figure 4d, with the calculated rise and fall time being 2.6 and 0.6 s, respectively. Figure 4e shows the device's photocurrent as a function of laser power, with the responsivity of 1.72 µA/W at *V_DS_* = −1 V. Figure 4f shows the photocurrent of the device shown in Figure 4c as a function of *V_DS_*_._ Importantly, the devices show a photocurrent at *V_DS_* = 0 V indicating that they have a built‐in potential at the MoSe_2_‐WSe_2_ junction which drives a photovoltaic effect. These results illustrate the functional device performance of TMD heterojunctions that were characterized and fabricated using image analysis and alignment, highlighting the utility of high‐throughput methodologies for 2D material device integration.

**Figure 5:**
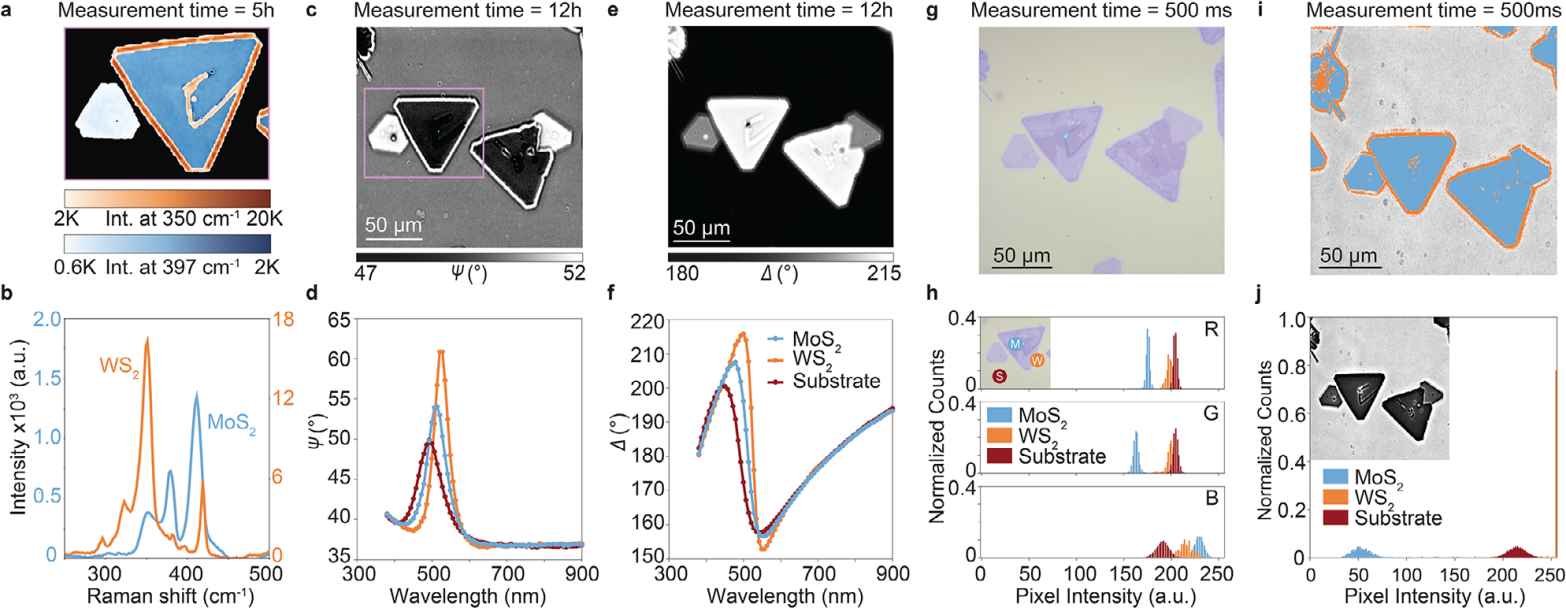
a) Raman intensity map of MoS_2_‐WS_2_ heterostructures at 350 and 397 cm^−1^, the color bar shows counts per acquired spectrum. b) Raman characterization showing spectra of MoS_2_ and WS_2_ regions. c) Δ map of MoS_2_‐WS_2_ heterostructures at 500 nm measured in rotating compensator ellipsometry (RCE) imaging mode. d) Δ as a function of wavelength as measured for S) the silicon substrate, W) WS_2_ region, and M) MoS_2_ region shown in the inset of (h). e) Ψ map of MoS_2_‐WS_2_ heterostructures at 500 nm measured in rotating compensator ellipsometry (RCE) imaging mode. f) Δ as a function of wavelength as measured for S) the silicon substrate, W) WS_2_ region, and M) MoS_2_ region shown in the inset of (h). g) Optical microscopy of lateral MoS_2_‐WS_2_ heterostructures on SiO_2_/Si. h) Histogram of an RGB image shown in (g) for R, G, and B pixels of i) MoS_2_, ii) WS_2_ and iii) substrate regions as shown in the inset. i) Image segmentation used to detect MoS_2_ and WS_2_ material from an ellipsometry image at optimized contrast settings of MoS_2_‐WS_2_ heterostructures (475 nm, C = 45°, P = 55° and A = 140°). j) Histogram of a grayscale image with optimized contrast for pixels of single layer and bilayer MoS_2_, ii) WS_2_ and iii) substrate regions as shown in the inset.

To further demonstrate the benefits of ECM for lateral heterostructure characterization, we evaluate sulfide based heterostructures; MoS_2_‐WS_2_. Raman spectroscopy (**Figure** **5**a,b) of a pair of as‐grown crystals on an oxidized Si substrate reveals a central island of MoS_2_ with a typical width of≈50 µm. The Raman map in Figure 5a shows the central island is surrounded by WS_2_ in a lateral heterostructure. RCE‐mode imaging ellipsometry of the same region, as described above, was used to determine *Δ* and *Ψ* as a function of wavelength (Figure 5c–f). We focus our discussion here on the larger of the two triangular flakes highlighted in the square in Figure 5c in which the central island appears to be bilayer MoS_2_. Both *Δ* and *Ψ* show significant changes in the optical properties of the flakes as a function of layer number and material, which can be exploited in ECM imaging. An example of ECM imaging of the same region is given at λ = 513 nm in the inset of Figure 5j. The pixel intensity histogram is clearly separated into three categories, with the lower intensity regions attributed to MoS_2_ and the highest intensity regions attributed to WS_2_. When these thresholds are applied to the segmented ECM image (Figure 5i) the outer regions of WS_2_ can be clearly observed. By comparison, conventional white light reflection microscopy (Figure 5g) offers low contrast due to overlapping RGB intensity histograms (Figure 5h) for the different regions. We note that while the contrast under conventional white light reflection microscopy is low, it is not negligible because of optical interference effects between the 2D layers and the oxidized Si substrate.^[^ ^58^ ^]^ The contrast in white light reflection microscopy is highly dependent on the dielectric environment, and any modification to the substrate or surrounding medium can eliminate this contrast, limiting the usefulness of white light reflection microscopy. ECM, however, is more versatile providing contrast with a broader range of surrounding media. To demonstrate this, we spin coat a MoS_2_‐WS_2_ heterostructure on oxidized Si with a capping layer of PMMA (Figure S4, Supporting Information). The presence of the PMMA layer renders the WS_2_ indistinguishable from the substrate in brightfield reflection microscopy, but the WS_2_ is still clearly observable under ECM.

## 3 Conclusion

This work presents a high throughput ECM‐based method for identification and characterization of the material distribution within randomly nucleated 2D semiconductor MoSe_2_‐WSe_2_ lateral heterostructures. As a wide‐field microscopy technique where each field of view is captured in less than 1 s, ECM offers significant advantages over point‐by‐point characterization, while maintaining high material contrast. By automating the process using computer vision algorithms it was possible to generate a database of over 1300 localized and characterized individual 2D TMD lateral heterostructures. The presented technique can provide datasets that are sufficiently large for statistical analysis of 2D material growth and resulting properties. By integrating machine‐readable fiducial markers onto the substrate, the position of each randomly nucleated feature can be determined for device integration, representing a scalable method of advanced 2D material device manufacturing. These results highlight the benefits of ECM combined with image processing as a reliable and fast technique for large‐area characterization and device integration of 2D materials.

## Conflict of Interest

The authors declare no conflict of interest.

## Supporting information



Supporting Information

## Data Availability

The data that support the findings of this study are openly available in the Cambridge University data repository at https://doi.org/10.17863/CAM.116278.
