# Supplementary material for: High‐Throughput Ellipsometric Contrast Microscopy of Lateral 2D Heterostructures for Optoelectronics
Source: Small Methods. 2025 Jul 9;10(2):2500437. doi: 10.1002/smtd.202500437 (PMC12825334; doi:10.1002/smtd.202500437)
Supplement: Supplementary file 1 — Supporting Information [file SMTD-10-2500437-s001.pdf]

Supporting Information for: High-Throughput Ellipsometric Contrast Microscopy of Lateral 2D Heterostructures for Optoelectronics

Teja Potočnik[1], Oliver Burton[1], Suman K. Chakraborty[2], Purbasha Ray[2], Ralf Mouthaan[3], Peter J. Christopher[4], Zeinab Tirandaz[1], Xiaofan Lin[1], Hannah Joyce[1], Stephan Hofmann[1], Prasana K. Sahoo[2], Jack A. Alexander-Webber[1*]

[1]Department of Engineering, University of Cambridge, 9 JJ Thompson Avenue, Cambridge CB3 0FA, United Kingdom

[2] Materials Science Center, Indian Institute of Technology Kharagpur, Kharagpur, West Bengal, 721302, India

[3]Centre of Light for Life, University of Adelaide, North Terrace, Adelaide SA 5005, Australia

[4]Department of Engineering, University of Nottingham, Nottingham, NG7 2RD, United Kingdom

Email: jaa59@cam.ac.uk

Methodology:

Heterostructure growth: $MoSe_2$-WSe_2 heterostructures were grown using a one-pot chemical vapor deposition (CVD) synthesis approach. A mixture of heterogeneous solid source (MX_2, M=Mo, W and X= Se, S) is used during the continuous growth of monolayer lateral junction heterostructures while changing the composition of the reactive gas environment in the presence of water vapour. The process enables selective control of nucleation on the substrate, water-induced oxidation and volatilisation of each transition metal precursor, resulting in sequential-edge epitaxy of $MoSe_2$-WSe_2 heterostructures. For more information, see reference [1].

Ellipsometry: Ellipsometry measurements were taken using a commercial Accurion EP4 ellipsometer in RCE configuration, using a 20× objective. All measurements were conducted at room temperature under ambient conditions unless otherwise specified. The model fitting was performed with $MoSe_2/SiO_2/Si$ and $WSe_2/SiO_2/Si$ models using the Tauc-Lorentz function to describe the dielectric function, $\varepsilon = \varepsilon_1 + i\varepsilon_2$ of $MoSe_2$ and WSe_2. The imaginary part of the dielectric function is given by:

$$\varepsilon_2 = \frac{A\,E_0 C\,\left(E - E_g\right)^2}{(E^2 - E_0^2)^2 + C^2 E^2}\frac{1}{E} \quad for\ E > E_g$$

$$\varepsilon_2 = 0\ for\ E \leq E_g$$

Where A is the amplitude of the peak, E_0 the peak position, C the half-width of the peak and E_g the bandgap. The real part ε_1 is derived from the Kramers-Kronig relations [2]:

$$\varepsilon_1 = \varepsilon_1(\infty) + \frac{AC}{\pi \xi^4}\frac{\alpha_{ln}}{2\alpha\,E_0}\ln\left(\frac{E_0^2 + E_g^2 + \alpha E_g}{E_0^2 + E_g^2 - \alpha E_g}\right)$$

$$-\frac{A}{\pi\xi^4}\frac{\alpha_{tan}}{E_0}\left(\pi - tan^{-1}\left(\frac{2E_g + \alpha}{C}\right) + tan^{-1}\left(\frac{-2E_g + \alpha}{C}\right)\right)$$

$$+ 2\frac{AE_0}{\pi\xi^4\alpha}E_g(E^2 - \gamma^2)\left(\pi + 2\,tan^{-1}\left(2\frac{\gamma^2 - E_g^2}{\alpha C}\right)\right)$$

$$-\frac{AE_0 C}{\pi\xi^4}\frac{E^2 + E_g}{E}\ln\left(\frac{|E - E_g|}{E + E_g}\right)$$

$$+\frac{2AE_0 C}{\pi\xi^4}E_g\ln\left(\frac{|E - E_g|(E + E_g)}{\sqrt{(E_0^2 - E_g^2)^2 + E_g^2 C^2}}\right)$$

where

$$\alpha_{ln} = (E_g^2 - E_0^2)E^2 + {E_g}^2C^2 - E_0^2(E_0^2 + 3E_g^2)$$

$$\alpha_{tan} = (E^2 - E_0)(E_0^2 + E_g^2) + E_g\, C^2$$

$$\xi^4 = (E^2 - \gamma^2)^2 + \frac{\alpha^2 C^2}{4}$$

$$\alpha = \sqrt{4E_0^2 - C^2}$$

$$\gamma = \sqrt{E_0^2 - \frac{C^2}{2}}$$

We initially measured the bare substrate to determine the thickness of the SiO_2 to use as a fixed parameter when fitting the TMD layers. For each material, $MoSe_2$ and WSe_2, we fit the thickness t, and a set of peak parameters: A, E_0, C, E_g and $\varepsilon_1(\infty)$. We expect to see three peaks in this wavelength range for monolayer $MoSe_2$ and four peaks for WSe_2, which are associated with different excitonic transitions [3]. The best-fit parameters of the Tauc-Lorentz model of $MoSe_2$ and WSe_2 are shown in Table 1, and the models plotted in Figure S1.

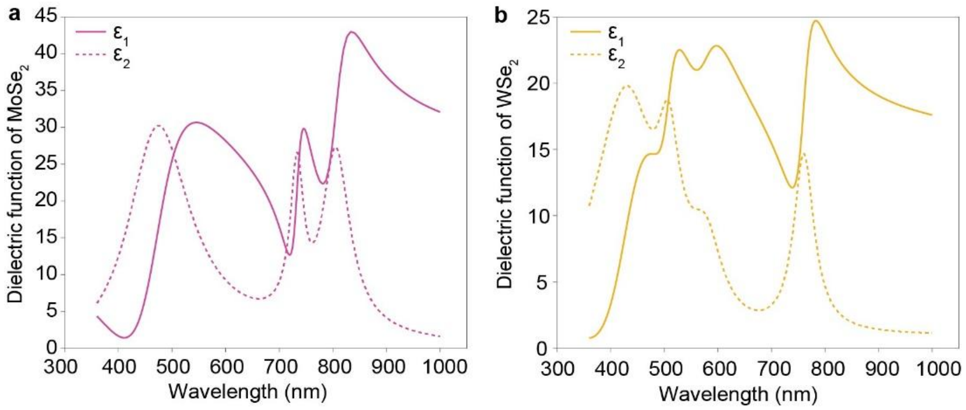

Figure S1: Real and imaginary part of the dielectric function of $MoSe_2$ (a) and WSe_2 (b).

Wavelength-dependent ellipsometric contrast mapping (ECM) was conducted with an Accurion EP4 Ellipsometer at 20× magnification. Polariser and analyser angles were determined by maximising the magnitude of the Weber contrast of a $MoSe_2$ region relative to WSe_2 region (see Figure 2). Polariser, analyser and compensator position control was enabled with an Accurion ellipsometer application programming interface (API). Monochrome images were then acquired for a range of wavelengths of incident light. For 450 monochrome images at different wavelengths (each ~1 MP resolution), this took approximately 5 minutes, including focal line scanning, with a single image taking approximately 500 ms. The comparison of ECM to other techniques is shown in Table 2

Table 1: Fitted parameters for the $MoSe_2$ and WSe_2 dielectric modelled from Δ and Ψ measurement as a function of λ in Figure 1e and f.

	**$MoSe_2$**	**WSe_2**
**Thickness (nm)**	0.69	0.62
$\varepsilon_1(\infty)$ (eV)	14.41	6.92
A_1 (eV)	44.12	106.21
E_{01} (eV)	2.62	2.83
C_1 (eV)	0.78	0.94
E_{g1} (eV)	0.71	1.70
A_2 (eV)	15.65	3.47
E_{02} (eV)	1.69	2.44
C_2 (eV)	0.07	0.24
E_{g2} (eV)	1.18	0.40
A_3 (eV)	3.36	10.73
E_{03} (eV)	1.54	2.14
C_3 (eV)	0.12	0.28
E_{g3} (eV)	0.09	1.28
A_4 (eV)	N/A	4.20
E_{04} (eV)	N/A	1.63
C_4 (eV)	N/A	0.10
E_{g4} (eV)	N/A	0.69
RMSE	4.78	4.17

Table 2: Comparison of large-area mapping techniques for thin-film characterisation for a ~200×200μm² area.

	**Raman spectroscopy (raster scanning laser spot)**	**Brightfield optical microscopy**	**Imaging ellipsometry (RCE mode with pixel-by-pixel model fitting)**	**ECM**
**Speed**	Acquisition ≈ 4h<br>Analysis[1] ≈ 5min	Acquisition ≈ 1s<br>Analysis[2] ≈ 5s	Acquisition ≈ 12h<br>Analysis[3] ≈ 72h	Acquisition ≈ 1s<br>Analysis[2] ≈ 5s
**Weber contrast ($MoSe_2$-WSe_2)**	9 (Figure 1d @ 250 cm^{-1})	0.05 (Figure 3, G channel)	0.175 (Figure 1f, Ψ @ λ = 500 nm)	0.8 (Figure 2e)
**Substrate requirements**	Low-fluorescence	Substrate providing optical contrast (e.g., SiO_2/Si for interference)	Reflective, flat	Reflective, flat
**Rate-limiting step**	Point-by-point scanning; data processing (e.g., peak fitting)	Image interpretation if contrast is low; reliance on contrast mechanism	Polariser/compensator rotation, model fitting for quantitative maps	Optimisation of settings requires prior sample knowledge

[1]Includes baseline correction, fitting peaks, generating spectral maps from each spectra.
[2]Based on simple thresholding of intensity values.
[3]Based on pixel-by-pixel optical model fitting to extract quantitative parameters like layer thickness and refractive indices across the mapped area.

Raman spectroscopy: Raman spectroscopy was performed using a 532 nm laser excitation with an exposure time of 1 s, laser power in the order of 1 mW, a grating of 1800 l/mm, and a 50× objective in a Renishaw inVia Raman system.

Image processing: Optical microscopy and ellipsometry images were analysed in MATLAB to automatically locate and identify regions of interest on a sample. The images were first processed by i) performing image segmentation based on the pixel intensity of specific materials, ii) converting the image to grayscale, iii) removing small areas below a threshold size from the image and iv) morphologically closing the image by dilating the features using a square structuring element.

After desired regions have been successfully isolated from the image, the *regionprops* command locates the regions of interest. This is achieved based on parameter filtering, such as region area, length, width, orientation, circularity, and solidity.

The centre-to-edge ($MoSe_2$) and edge-to-edge (WSe_2) width of each respective material were determined using the *regionprops* function in MATLAB that finds the equivalent radius, which is the radius of a circle with the same area as the detected feature. This was used to include the detection of flakes that are not perfect shapes and/or are being partially covered. The

dimensions of the inner and outer flakes were determined based using their central coordinates as a reference. For every $MoSe_2$ flake region that was identified from the segmented images, we correlate it to the WSe_2 flakes by finding the flake with the exact same central coordinate.

Device fabrication: The samples with $MoSe_2$-WSe_2 on SiO_2/Si substrates were spin-coated using PMMA 495 A8 at 4000 rpm and then heated at 120° C for 3 minutes. The device patterns were exposed using electron beam lithography in the Raith EBPG 5200 EBL system. Following development in 15:5:1 IPA:MIBK:MEK solution for 25 s, the contacts were deposited using the Kurt J. Lesker PVD 200 Pro electron beam evaporator using 8 nm of Ti at 0.1 Å/s, followed by a 5 min wait period before depositing 60 nm of Au at 1 Å/s on top. Finally, the samples were cleaned in acetone and IPA to remove the resist.

Electrical characterisation: Electrical characterisation of heterostructure devices was performed using a probe station connected to a Keithley 4200-SCS semiconductor characterisation system, as well as the custom optoelectronic characterisation system using ThorLabs Multi-Channel Fibre Coupled Laser source and a Keithley 2635B System SourceMeter. White light illumination for the data in Figure 4a was supplied by a 3200 K halogen lamp with a power density of ~30 mW cm^{-2}. All other photocurrent measurements were measured using laser excitation at λ = 685 nm and a focused spot diameter of approximately 4 μm. All optoelectronic measurements were carried out at room temperature under ambient conditions unless otherwise specified.

Optimising Imaging Conditions: Figure S2 shows different contrast between $MoSe_2$ and WSe_2 material for different ellipsometry settings.

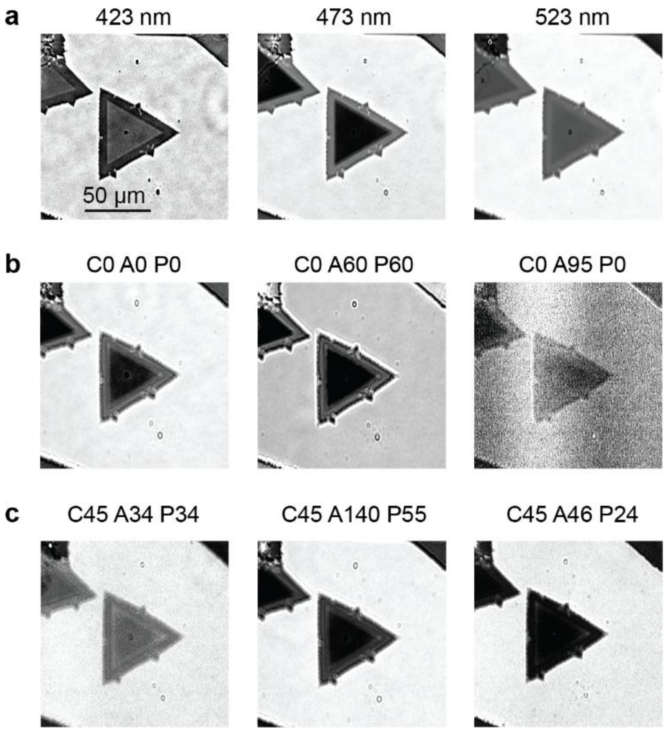

Figure S2: Example ECM images of $MoSe_2$-WSe_2 heterostructures at AOI = 41° for different (a) wavelengths with P = A = C = 0°, (b) P and A angles at C = 0° and (c) P and A angles at C = 45° for λ = 475 nm. The central column of images represents the conditions for optimal contrast within a parameter sweep.

Optimising Layer Number Contrast: Growth processes were tailored to maximise monolayer coverage in the sample, as TMDs such as $MoSe_2$, MoS_2, WSe_2 and WS_2 exhibit desirable direct bandgaps and valley structure in monolayer form. TMDs lose this desirable bandstructure at higher layer numbers. Consequently, it is important to distinguish monolayer regions from regions of higher layer number. Here we demonstrate the ECM is capable of discerning layer number in lateral heterostructure TMDs, comparable to its previously demonstrated capability of determining layer number in multilayer graphene [4], [5]. Figure S3 shows the wavelength-dependent contrast between monolayer (1L) and bilayer (2L) $MoSe_2$ (Figure S3a) and MoS_2 (Figure S3d) at P = A = C = 0° and AOI = 41°. Layer-number contrast, which we define as $(Int_{1L} - Int_{2L})/Int_{2L}$, where Int_{1L} and Int_{2L} is the average intensity of monolayer and bilayer of the same material, is maximised for λ = 494 nm and 533 nm for $MoSe_2$ and MoS_2 respectively, with example ECM images shown in Figure S3b and Figure S3e respectively. Plotting a histogram of pixel intensities (Figure S3c, f) demonstrates that there is a clear separation between monolayer and bilayer regions, which can be segmented using thresholding techniques described in the main manuscript. We find the wavelength of peak intra-material layer contrast is distinct from that of peak inter-material contrast. This could motivate the use of dual-wavelength ECM modalities to gain further high-throughput sample characteristics.

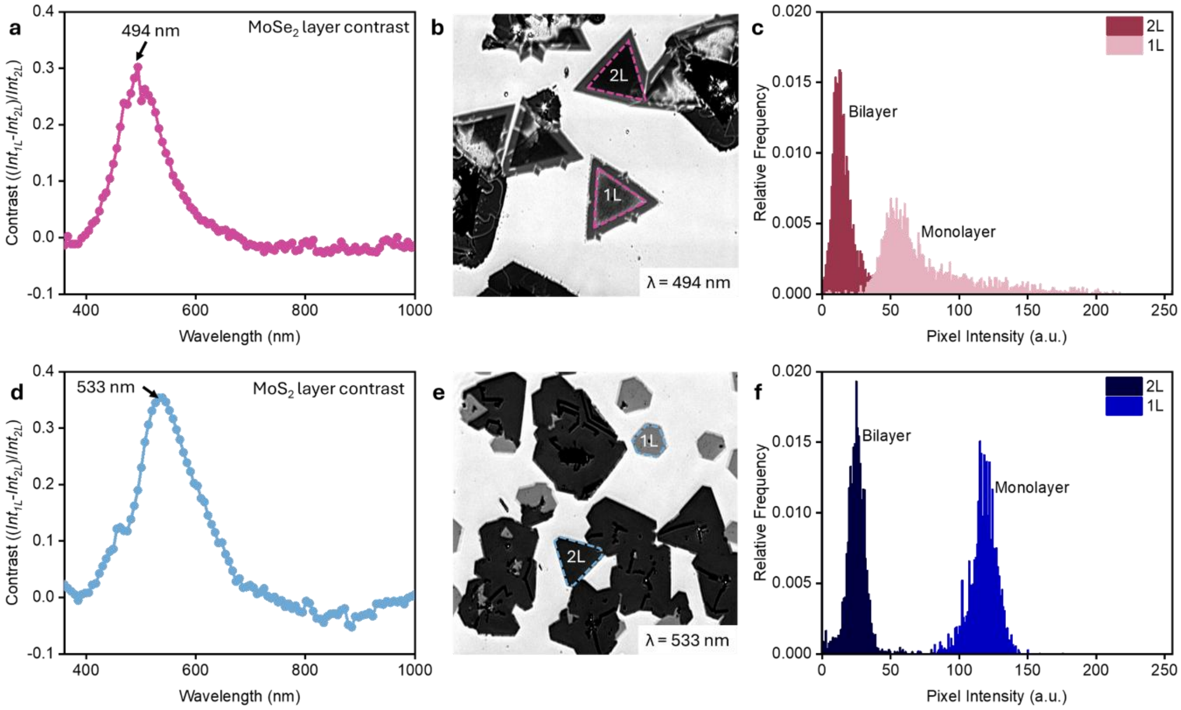

Figure S3: (a) Wavelength dependent contrast between monolayer and bilayer $MoSe_2$ and (b) an example ECM image at the wavelength of peak contrast. (c) Histogram of pixel intensity for monolayer (1L) and bilayer (2L) $MoSe_2$ regions labelled in (b) showing well separated layer dependent distributions. (d) Wavelength dependent contrast between monolayer and bilayer MoS_2 and (e) an example ECM image at the wavelength of peak contrast. (f) Histogram of pixel intensity for monolayer (1L) and bilayer (2L) regions of MoS_2 labelled in (e).

ECM on non-contrast-enhancing substrates: As the samples were grown directly on Si substrates which have a layer of thermally grown SiO_2 (t_{SiO2} = 276.7 nm), the brightfield reflection microscopy images with white light illumination, such as those shown in Figures 1a, and 5g and Figure S4a, benefit from contrast enhancement due to optical interference effects of incident light [6]. For comparison, we now reduce that contrast enhancement effect by adding an additional layer to the substrate, namely, by spin coating ~500 nm of PMMA 495k, followed by drying on a hotplate. This combination of a change in thickness of the layer stack and the dielectric constant of PMMA, makes the outer WS_2 of a monolayer MoS_2-WS_2 heterostructure indistinguishable from the substrate under brightfield reflection microscopy with white light illumination (Figure S4b). By imaging the same sample using ECM, we obtain clear contrast between the MoS_2, WS_2, and the substrate (Figure S4c), confirming the versatile benefits of ECM for high-throughput inspection of 2D material heterostructures on a wide range of substrates.

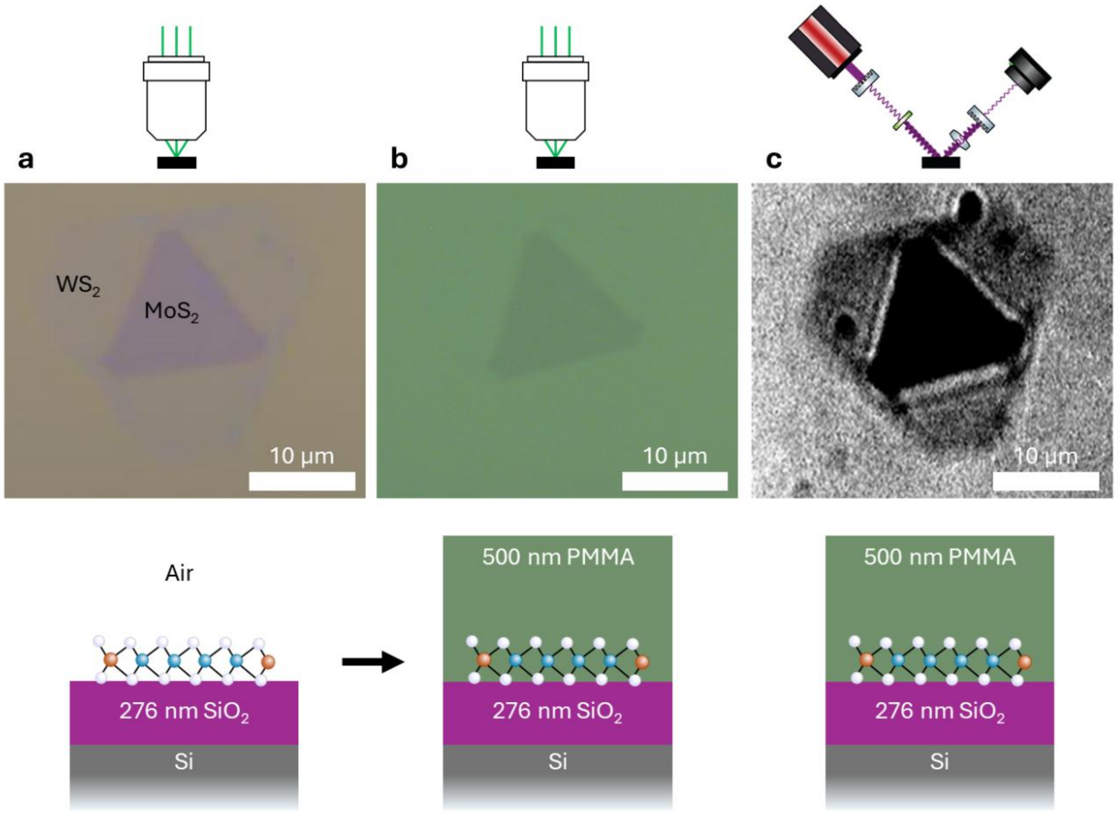

Figure S4: Brightfield reflection microscope image of a monolayer lateral MoS_2-WS_2 heterostructure under white light illumination before (a) and after (b) spin coating 500nm of PMMA 495k. (c) The same area of the sample after PMMA coating imaged under ECM for λ = 635 nm, P = A = 90°, C = 0° and AOI = 41°.

References

[1] P. K. Sahoo, S. Memaran, Y. Xin, L. Balicas, and H. R. Gutiérrez, "One-pot growth of two-dimensional lateral heterostructures via sequential edge-epitaxy," *Nature*, vol. 553, no. 7686, pp. 63–67, Jan. 2018, doi: 10.1038/nature25155.

[2] H. Fujiwara, *Spectroscopic Ellipsometry: Principles and Applications*. Tokyo, Japan: Maruzen Co. Ltd, 2003.

[3] Y. Li *et al.*, "Measurement of the optical dielectric function of monolayer transition-metal dichalcogenides: MoS_2, $MoSe_2$, WS_2, and WSe_2," *Phys. Rev. B*, vol. 90, no. 20, p. 205422, Nov. 2014, doi: 10.1103/PhysRevB.90.205422.

[4] P. Braeuninger-Weimer *et al.*, "Fast, noncontact, wafer-scale, atomic layer resolved imaging of two-dimensional materials by ellipsometric contrast micrography," *ACS Nano*, vol. 12, no. 8, pp. 8555–8563, 2018, doi: 10.1021/acsnano.8b04167.

[5] T. Potočnik *et al.*, "Fast Twist Angle Mapping of Bilayer Graphene Using Spectroscopic Ellipsometric Contrast Microscopy," *Nano Lett.*, vol. 23, no. 12, pp. 5506–5513, Jun. 2023, doi: 10.1021/acs.nanolett.3c00619.

[6] P. Blake *et al.*, "Making graphene visible," *Appl. Phys. Lett.*, vol. 91, no. 6, p. 63124, 2007, doi: 10.1063/1.2768624.
